# Identifying Universal Fish Biomarker Genes in Response to PCB126 Exposure by Comparative Transcriptomic Analyses

**DOI:** 10.3390/cimb46080466

**Published:** 2024-07-23

**Authors:** Ira Agrawal, Ai Qi Lee, Zhiyuan Gong

**Affiliations:** 1 Department of Biological Sciences, National University of Singapore, Singapore 117558, Singapore; phsia@nus.edu.sg (I.A.); aiqi001@e.ntu.edu.sg (A.Q.L.); 2 Department of Physiology, National University of Singapore, Singapore 117593, Singapore

**Keywords:** zebrafish, medaka, tilapia, guppy, PCB126, biomarker

## Abstract

Water pollution remains a major environmental concern, with increased toxic by-products being released into water bodies. Many of these chemical contaminants persist in the environment and bio-accumulate in aquatic organisms. At present, toxicological tests are mostly based on laboratory tests, and effective methods for monitoring wild aquatic environments remain lacking. In the present study, we used a well-characterized toxic chemical, 3,3′,4,4′,5-polychlorinated biphenyl (PCB126), as an example to try to identify common biomarker genes to be used for predictive toxicity of this toxic substance. First, we used two laboratory fish models, the zebrafish (*Danio rerio*) and medaka (*Oryzias latipes*), to expose PCB126 to obtain liver transcriptomic data by RNA-seq. Comparative transcriptomic analyses indicated generally conserved and concerted changes from the two species, thus validating the transcriptomic data for biomarker gene selection. Based on the common up- and downregulated genes in the two species, we selected nine biomarker genes to further test in other fish species. The first validation experiment was carried out using the third fish species, Mozambique tilapia (*Oreochromis mossambicus*), and essentially, all these biomarker genes were validated for consistent responses with the two laboratory fish models. Finally, to develop universal PCR primers suitable for potentially all teleost fish species, we designed degenerate primers and tested them in the three fish species as well as in another fish species without a genomic sequence available: guppy (*Poecilia reticulata*). We found all the biomarker genes showed consistent response to PCB126 exposure in at least 50% of the species. Thus, our study provides a promising strategy to identify common biomarker genes to be used for teleost fish analyses. By using degenerate PCR primers and analyzing multiple biomarker genes, it is possible to develop diagnostic PCR arrays to predict water contamination from any wild fish species sampled in different water bodies.

## 1. Introduction

Water pollution by toxic by-products from human activities is an important concern, as many of the pollutants persist and bio-accumulate in living organisms and the environment [1]. It is estimated that 300–400 million tons of industrial wastes are dumped into water bodies every year [2]. These chemicals are discharged into the water system, which has major implications on the health of not only the aquatic living organisms but also humans through food chain and water activities. An example underscoring the magnitude of the concern and the importance of monitoring aquatic toxicity is the Minamata disaster caused due to the release of methylmercury in industrial waste water, which caused death and injury to thousands due to mercury poisoning [3].

Although various physicochemical tests are available to detect water pollutants, they generally do not provide information on the biological impact of pollutants on living organisms and potential health risks. Thus, it is necessary to develop convenient biomonitoring technology to analyze environmental pollution and its effects on living organisms and the environment. In the aquatic environment, several aquatic organisms, such as algae, water fleas, mollusks and fish, have been commonly used for biomonitoring [4,5,6]. In particular, teleost fish are a favorable system for aquatic biomonitoring, as they are a highly diversified group of vertebrates and live in a wide variety of aquatic environments, from freshwater lakes and rivers to different levels of the ocean. They are easily available and share great similarity with humans in gene expression and physiology activity; thus, fish could be a highly suitable and relevant system for monitoring aquatic environmental pollution. Embryo/larval tests for chemical toxicity based on developmental lethal and sub-lethal endpoints are currently used in laboratory settings for several small aquarium fish such as the zebrafish and medaka embryos [7,8], but standard methods for monitoring wild aquatic environments remain lacking.

For biomonitoring systems, biomarker genes are frequently used, as their expression can be conveniently and quantitatively analyzed [9]. As compared to morphological and physiological changes, molecular markers can be more sensitive to environmental changes and can act as an early warning system [10,11]. Some biomarker genes specific to certain types of chemicals have been well established, and bioassays have been developed. For example, increased *cyp1a* and *vtg1* expressions have been widely used as indicators of the presence of polycyclic aromatic hydrocarbons and estrogenic compounds, respectively [9,12,13]. However, in view of the vast number of environmental pollutants, the number of characterized and verified biomarker genes is rather limited. Thus, there is an urgent need to screen potential biomarkers genes for more toxic chemicals.

To rapidly identify potential biomarker genes for monitoring chemical contamination, we proposed to carry out a transcriptomic comparison of multiple fish species to identify common dysregulated genes by the same chemical. The xenobiotic chemical metabolism, specifically aromatic hydrocarbon-mediated toxicity via the aryl hydrocarbon receptor (AhR) pathway, which regulates the cytochrome P450 complex enzymes mediating xenobiotic oxidation, is well studied and thus was chosen for this proof-of-concept study [14]. A category of strong AhR agonists is the polychlorinated biphenyls (PCBs), which have been shown to cause a wide variety of effects, including skin lesions, neurotoxicity, reproductive toxicity, and damage to immune function and cardiovascular function [15,16]. They are also potent endocrine disruptors and activators of hepatic enzymes such as cytochrome P450 complex enzymes. Here, we used a typical inducer of the AhR pathway, a co-planar polychlorinated biphenyl, namely 3,3′,4,4′,5-polychlorinated biphenyl (PCB126), as an example to identify common responsive biomarker genes in multiple fish species. PCB126 is a highly chlorinated and lipophilic biphenyl that is resistant to degradation, toxic, and bio-accumulative, particularly in fatty tissue [17,18]. Air contamination is a major source of PCB126 exposure, but exposure can also occur through the diet via contaminated food like fish and dairy products [18]. Exposure has been shown to cause adverse effects in various systems, for example, cardiovascular diseases [19], diabetes mellitus [20], liver diseases [21], and alterations in inflammatory and neurological pathways [19,22,23].

In the present study, the genome-wide identification of liver genes responsive to PCB126 exposure was first carried out in two popular laboratory model fish, i.e., zebrafish (*Danio rerio*) and medaka (*Oryzias latipes*), which diverged early in teleost evolution. Potential biomarker genes identified from the two fish species were then validated in a third fish species, Mozambique tilapia (*Oreochromis mossambicus)*. The validated biomarker genes from all the three species were then selected to use to design universal degenerate PCR primers that are potentially suitable for most, if not all, teleost species, which were then tested in a fourth fish species, guppy (*Poecilia reticulata*), together with the other three species. Here, we present our test data to indicate the feasibility of this strategy to identify common biomarker genes for toxic chemicals and to develop common molecular assays using universal PCR primers for potentially all fish species in different aquatic environments.

## 2. Materials and Methods

### 2.1. Fish Maintenance

Wild-type zebrafish (*Danio rerio*), Hd-rR wild-type strain of medaka (*Oryzias latipes*), wild-type tilapia fingerlings (*Oreochromis mossambicus*, ~10 cm in length), and wild-type guppies (*Poecilia reticulata*) were used in experiments conducted in the fish facility of Department of Biological Sciences, National University of Singapore. Adult wild-type zebrafish (3–5 months old) and wild-type guppies (3–4 months old) were obtained from the Mainland Tropical Fish Farm, Singapore. Laboratory Hd-rR wild-type strain of medaka were obtained from National BioResource Project (NBRP Medaka), Japan, and maintained in the laboratory. Wild-type tilapia fingerlings (3–4 months old) were obtained from Temasek Life Sciences Laboratory Limited, Singapore. The fish were acclimatized to laboratory conditions for 2 weeks before experiments. In the laboratory, the fish were maintained in a 14:10 h light/dark cycle at 28 ± 2 °C in sea salt solution (60 mg of Instant Ocean salt per liter of distilled water). All maintenance and experimental protocols with fish were approved by the Institutional Animal Care and Use Committee (IACUC) of National University of Singapore, Singapore.

### 2.2. Acute PCB126 Treatment

PCB126 (catalog number: C-126N, manufactured by AccuStandard, New Haven, CT, USA) was dissolved in dimethyl sulfoxide (DMSO). Then, 1000X stock solution was prepared and diluted to working concentration, 150 nM [24]. Standard 96 h acute toxicity exposure was conducted using 0.1% DMSO as the control in high-density polyethylene (HDPE) tanks of 5 L capacity. Male fish aged 3–5 months old were used for the acute exposure experiments. Zebrafish, medaka. and guppies were housed at a density of 10 fish per 5 L tank, with separate tanks for each species. Twenty fish per treatment group (controls and acute exposed fish) were used for each of the three species of zebrafish, medaka, and guppy. Tilapia fingerlings, which were larger and more aggressive, were housed at 5 fish per 5 L tank, with 10 fish per treatment group. Since tilapia fingerlings were much larger in size than the other fish species, toxicity treatment was carried out at two concentrations: 1X and 1.5X the concentrations used for zebrafish and medaka treatment, i.e., at 150 nM and 225 nM of PCB126.

Half of the media was renewed every 24 h to maintain water quality and chemical concentration, which was stagnant during the acute exposure experiment. Water quality standards were monitored daily using the HANNA multiparameter photometer (item # HI83200, manufacturer: HANNA Instruments, Woonsocket, RI, USA) and relevant LaMotte testing kits. The water quality parameters were maintained at 7.1–7.3 pH, 6.5–7.5 mg/L dissolved oxygen, 15–20 ppm alkalinity, and less than 0.02 mg/L ammonia. The fish were not fed during the experiment. On the completion of the acute toxicity exposure, the fish were euthanized in ice water, and liver samples were collected, weighed, and snap-frozen in liquid nitrogen and stored at −80 °C for subsequent use.

### 2.3. RNA Sequencing

The livers of 6 fish per treatment tank were pooled for both medaka and zebrafish, creating two biological replicate samples for each treatment and control group (2 tanks/treatment group were used). RNA was extracted from pooled liver samples using TriZol^®^ reagent (Invitrogen, Carlsbad, CA, USA) and sent to BGI Tech Solutions Co., Hong Kong, for library preparation and RNA sequencing. Based on the protocol provided by BGI, high-quality RNA samples with an RNA integrity number (RIN) greater than 8 and 28S/18S ratio in the range of 1.2–2.0 were used to generate TruSeq^®^ libraries (Illumina, San Diego, CA, USA). The library was then sequenced on the Illumina HiSeq^®^ 2000 platform (Illumina, San Diego, CA, USA). The sequencing was paired-end, and the depth of the samples ranged from 67–96 million reads of 90 bp length.

### 2.4. Bioinformatic Analysis

#### 2.4.1. Quantifying Gene Expression

The RNA-seq reads from zebrafish and medaka were aligned to reference *Danio rerio* GRCz11 ENSEMBL 94 release and *Oryzias latipes* ASM223467v1 ENSEMBL 94 release [25]. cDNA transcripts and transcript abundance were quantified using Kallisto v.0.44.0 [26] with standard parameters and 100 bootstraps. Batch effects in the samples were removed using ComBat [27] from the sva R package [28], and these corrected-count data were used for further analysis.

#### 2.4.2. Homolog Mapping

Medaka-to-zebrafish homology information was taken from Ensembl Compara [29,30,31]. From this, 15,569 genes with one-to-one orthologs and high-confidence, best one-to-many homologs (with a minimum 25% sequence alignment) were selected. All medaka genes were mapped to their corresponding zebrafish homologs and used for further analysis. Similarly, we used Ensembl Compara to obtain 9878 high-confidence, best tilapia homologs for the 15,569 zebrafish genes. Homolog data from Ensembl Compara were also used for all other species comparisons.

#### 2.4.3. Differentially Expressed Genes (DEGs) and Functional Analyses

DEGs were identified using DESeq2 for treatment samples with respect to the DMSO control samples in medaka and zebrafish species separately. The resultant array of 15,569 homolog genes with log2 fold change values and adjusted *p*-values from the two species was used for comparison at gene-level.

GO (gene ontology) enrichment was performed using the enrichGO function of R package clusterProfiler. Gene-set enrichment analysis was performed using the PAGE algorithm from the R package piano [32,33]. The CP: curated canonical pathway collection of gene sets from MSigDB [33] consisting of 1329 sets was downloaded, and the human Entrez IDs used in the collection were converted to zebrafish gene IDs and filtered for gene sets with ≥4 zebrafish genes. Additionally, zebrafish GO sets from Ensembl were added to make the final number of gene sets analyzed 883, yielding a vector of Z-score for the degree of enrichment of each gene set in the sample per chemical treatment. Additional overrepresentation analysis was performed using DAVID [34,35] and WebGestalt [36,37].

### 2.5. Validation of Candidate Biomarkers

For validation, the chemical treatments and controls were conducted in duplicates to create two biological replicate samples per species for zebrafish, medaka, tilapia, and guppy, as described in Material and Methods Section 2.2: Acute PCB126 Treatment. A new batch of zebrafish and medaka, independent of those used for the initial transcriptomic analysis to identify candidate biomarkers, was used for the validation experiments to check the robustness of the candidate biomarkers. RNA was extracted from pooled liver samples using TriZol^®^ reagent, and reverse transcription was carried out using Transcriptor First Strand cDNA synthesis kit (Roche Applied Science, Basel, Switzerland). Expression of genes of interest was quantified by real-time qPCR using either species-specific primers or degenerate primers. RT-qPCR was carried out using the LightCycler^®^ 480 SYBR Green I kit and the LightCycler^®^ 480 Instrument II (Roche Applied Science).

### 2.6. PCR Primer Design

Tilapia homologs of the genes of interest were retrieved from Ensembl Compara, and cDNA sequence information for these genes was retrieved from the Ensembl database using the high-quality draft genome and assembly Orenil1.0 (GenBank assembly ID: GCA_000188235.1). NCBI Primer-BLAST [38] was used to design primers specific for tilapia genes. The primers were designed such that they had a melting temperature of 58–63 °C, at least two mismatches to unintended targets, and a product length in the range of 150–400 bp. Primer sequences are listed in Supplementary Table S1.

To design degenerate primers that would potentially target all teleost fish species, we used protein sequences of orthologous genes available for teleost fish. We selected 11 widely represented teleost species for which at least whole-genome raw assemblies are available: Amazon molly (*Poecilia formosa*), cave fish (*Astyanax mexicanus*), cod (*Gadus morhua*), fugu (*Takifugu rubripes*), medaka (*Oryzias latipes*), platyfish (*Xiphophorus maculatus*), spotted gar (*Lepisosteus oculatus*), stickleback (*Gasterosteus aculeatus*), tetraodon (*Tetraodon nigroviridis*), tilapia (*Oreochromis niloticus*), and zebrafish (*Danio rerio*). Homolog information for genes of interest (using zebrafish gene ID information as base) from all the above fish species was found using Ensembl Compara, and cDNA and protein sequence information was downloaded from Ensembl database.

The protein sequences were aligned using MUSCLE [39]. ClustalW files of the alignment were downloaded, and the j-CODEHOP tool was used to design COnsensus DEgenerate Hybrid Oligonucleotide Primers (CODEHOPs) capable of amplifying distantly related genes [40]. Each primer had a 3′ degenerate core and a 5′ consensus clamp. The 3′ degenerate core contains all the possible nucleotide combinations that code for 3–4 highly conserved amino acids in the protein alignment, allowing for broad specificity for distantly related target genes during PCR amplification. The 5′ consensus clamp sequence was built using the most common nucleotide in each codon for 5–7 amino acids immediately 5′ of the degenerate core, allowing for a robust amplification in the later cycles of PCR. Primers were selected for minimal degeneracy in the 3′ degenerate core and high clamp scores.

The data for the most common nucleotide used in codons for fish were derived from the Codon Usage database (http://www.kazusa.or.jp/codon/, accessed on 1 May 2018). The parameters used include clamp length (15–20 bp); core length (12 bp for 4 amino acids); strictness (0%); minimum amino acid conservation in core (80%); avoiding leucine, serine, and arginine from 3′ region (since these amino acids have a high number of codons); and low degeneracy of 8–64. Primer sequences are listed in Supplementary Table S2.

## 3. Results and Discussion

### 3.1. Transcriptomic Analyses of Zebrafish and Medaka in Response to PCB126 Exposure

Experimentally, zebrafish and medaka were exposed to 150 nM PCB126 to induce acute toxicity response. After 96 h of exposure, liver RNA was extracted and used for RNA sequencing. On average, zebrafish samples generated 67–78 million clean reads, out of which 66–67% could be mapped to the zebrafish genome. Medaka samples had a mapped-reads percentage of 61–63% out of 92–96 million clean reads. The number of expressed genes was around 20,000 for zebrafish and 15,000 for medaka samples.

**Figure 1:**
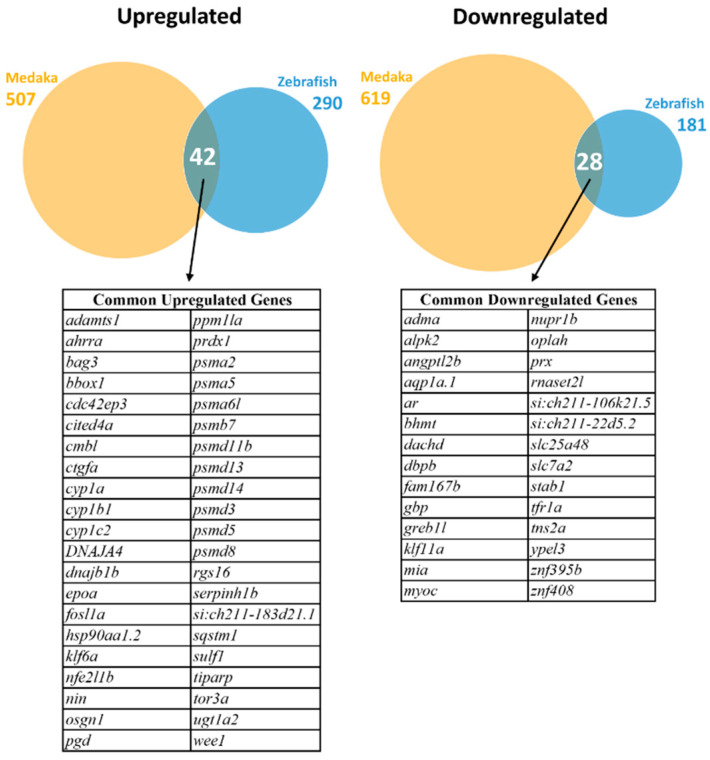
Identification of differentially expressed genes (DEGs) in zebrafish and medaka after PCB126 treatment. Upregulated DEGs are shown on the left and downregulated DEGs on the right. Venn diagrams of overlapping DEGs in the two fish species are presented for upregulated DEGs (**left**) and downregulated DEGs (**right**). The common upregulated and downregulated genes are listed below the Venn diagrams.

After comparison of the mapped zebrafish and medaka genes, 10,197 homologous genes were identified to be commonly expressed in the livers of both species and used for subsequent DEG analysis. DEGs were selected using cut-offs of absolute log2 fold change >1 and *p*-adjusted values < 0.05. As shown in Figure 1, a total of 471 DEGs (290 upregulated and 181 downregulated) were identified in zebrafish, while 1126 DEGs (507 upregulated and 619 downregulated) were identified in medaka. Of these significant DEGs, 42 common upregulated and 28 common downregulated genes were identified. These common DEGs are listed in Figure 1.

**Figure 2:**
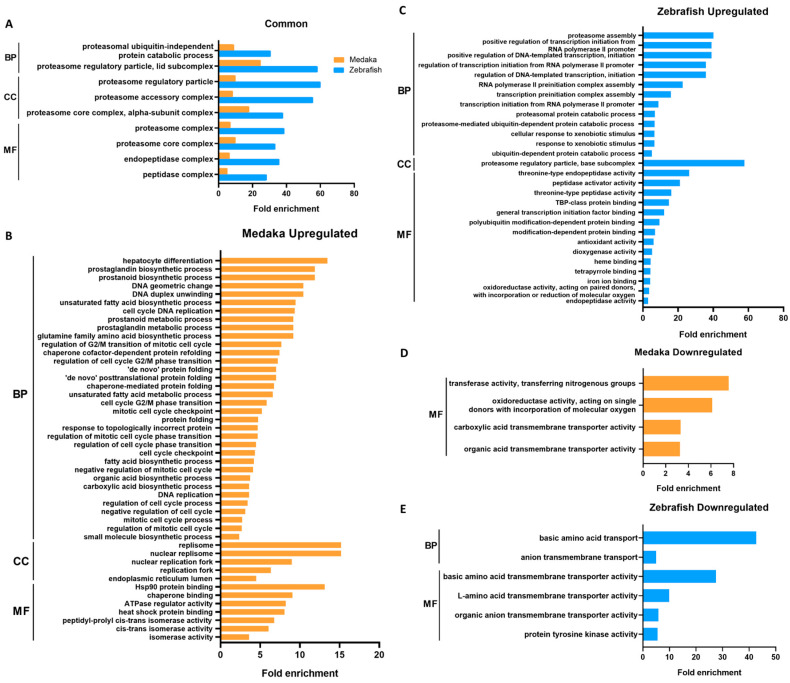
Conserved and unique GO terms significantly enriched in DEGs in PCB126-treated medaka and zebrafish, listed according to their fold enrichment values. (**A**) Common enriched GO terms in upregulated genes of PCB-treated medaka and zebrafish. (**B**–**E**) GO terms uniquely enriched in upregulated genes of medaka (**B**) and zebrafish (**C**) and in downregulated genes of medaka (**D**) and zebrafish (**E**). GO terms were filtered for Benjamini–Hochberg-corrected *p*-value < 0.05. BP: biological process; CC: cellular component; MF: molecular function.

The lists of DEGs were then annotated for biological function using gene ontology (GO). Significant GO terms (adjusted *p*-value < 0.05) in the DEGs of PCB-treated medaka and zebrafish are listed according to their GO categories and fold enrichment values in Figure 2. A total of nine GO terms were found to be commonly enriched in the upregulated DEGs of both species (Figure 2A). The most significantly enriched terms in both species are those related to proteasome protein degradation machinery. The common genes include proteasome structural and functional genes such as *psma*, *psmb*, *psmc*, and *psmd*. The rest of the enriched GO terms are also involved in proteolysis.

Upregulated and downregulated GO terms unique to each species are shown in Figure 2B–E. The enriched GO terms found only in upregulated medaka DEGs are mainly those involved in DNA replication, the mitotic cell cycle, protein folding response, and small molecule biosynthesis. Enriched GO terms from upregulated zebrafish DEGs are similar to the common terms (Figure 2A) but also include terms of transcription activity and xenobiotic response. Unique GO terms in both species seem to be related to small-molecule transport.

**Figure 3:**
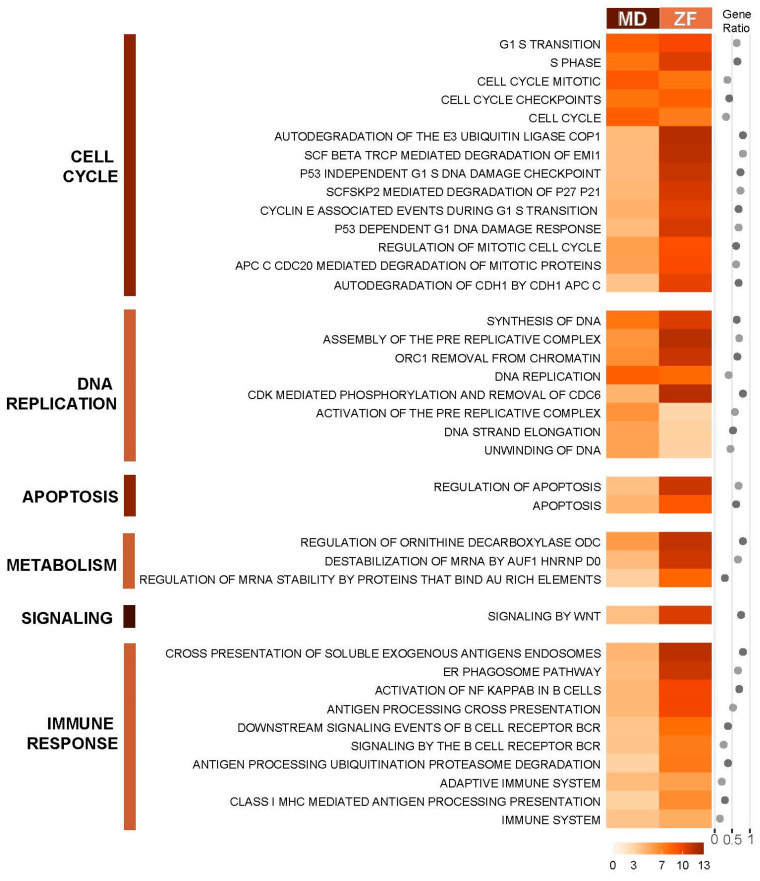
Conserved enriched canonical pathways in DEGs of medaka and zebrafish in response to PCB126. The canonical pathways were identified by PAGE analysis. Enrichment scores are presented along with gene ratio of number of genes found in our dataset by the total number of genes in the gene set. The darker the orange shade, the higher the enrichment score. Pathways are grouped based on Reactome pathway hierarchy from Reactome Knowledgebase. Pathways are filtered for a cut-off adjusted *p*-value of< 0.05.

The PAGE (Parametric Analysis of Gene-set Enrichment) algorithm [32] in the piano R package was also used to identify enriched pathways from the Reactome pathway database [41], as this method makes use of fold change and significance instead of using arbitrary cut-offs, allowing capture of the effect of coordinated small changes in gene expression. Identified conserved pathways in both medaka and zebrafish are grouped according to Reactome pathway hierarchy and presented in Figure 3. Despite only showing a relatively small number of commonly deregulated genes between the two fish species, PAGE demonstrates strong conservation of deregulated pathways in the PCB126 toxicity response between the two evolutionarily distant species. The common pathways identified across the two fish are pathways related to the cell cycle, DNA replication, DNA damage and apoptosis, protein degradation, and immune response, particularly pathways involved in B-cell response.

GO and PAGE analyses both show conserved significant enrichments of upregulated DEGs in gene sets and pathways regarding the breakdown of proteins in PCB126-exposed medaka and zebrafish livers. Proteasome-aided protein catabolism plays a significant homeostatic role in regulating the cellular environment by degrading ubiquitin-tagged proteins, unfolded or misfolded proteins, and/or proteins damaged by oxidative stress [42]. An increase in protein catabolism is an unsurprising effect of PCB126 exposure, as hundreds of genes are upregulated. Consistent with our transcriptomic data, PCB126 and its metabolites were also shown to produce reactive oxygen species [43], drastically increasing the risk and number of improperly folded proteins.

The cellular proliferation and apoptosis pathway enrichment suggests a carcinogenic effect of PCB126 on both fish species at the tested concentration. Indeed, PCB126 has carcinogenic abilities in the rat model, as demonstrated in a two-year chronic exposure study where female rats were induced with liver carcinoma, hepatocholangiomas, and bile duct hyperplasia [44]. Earlier, we also showed that PCB126 could accelerate hepatic tumorigenesis in a zebrafish liver-cancer model [45].

Immune response pathways, particularly those involving B-cell response, are also enriched in both medaka and zebrafish, in line with published studies on how PCB126 affects the immune system. Exposure to PCB126 has been shown to have both an immuno-activating effect and an immunosuppressive effect. PCB exposure leads to a decreased humoral response mediated by B cells in both fish and humans, with children showing a higher risk of infection and reduced protection upon vaccination [46,47,48]. Additionally, PCB126 exposure leads to a pro-inflammatory response, with increased nuclear factor kappa B (NFкB) expression and activity [49] and complement system stimulation [50].

In general, these comparative transcriptomic analyses for the two fish species indicate conserved and concerted biological responses to PCB126 treatment, thus validating the RNA-seq data to be used for selection of common biomarker genes for PCB126.

### 3.2. Selection and Validation of Selected Biomarker Genes in Multiple Teleost Species

Our ultimate goal is to identify common biomarker genes suitable in all fish species in order to monitor aquatic pollution from any available fish species in diverse water environments, including lakes, rivers, creeks, estuaries, oceans, etc. Here, we used PCB126 as an example toxicant chemical to develop a general approach for discovery of potential biomarker genes based on the transcriptomic analyses of two laboratory model fish, zebrafish and medaka. The 70 commonly deregulated genes identified in zebrafish and medaka provided a pool of potential biomarker genes for PCB126 exposure.

**Figure 4:**
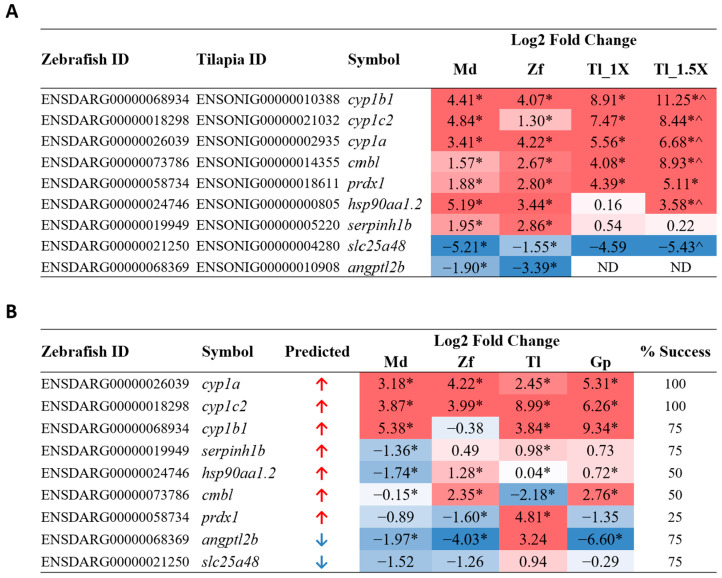
RT-qPCR validation of PCB126 candidate biomarker genes. (**A**) Validation in tilapia at 1X and 1.5X concentration, using tilapia-specific primers. Log2 fold change values for medaka and zebrafish were obtained from RNA-seq data and are presented alongside the tilapia qPCR values to show expression trends. * *p* < 0.05 when significance is tested against own species treated only with DMSO; ^ *p* < 0.05 when significance is tested against tilapia 1X group. Genes are listed according to tilapia log2 fold change values and predicted direction of deregulation. (**B**) Validation of PCB126 candidate biomarkers using common degenerate primers in all four fish species: medaka, zebrafish, tilapia, and guppy. Percentage success refers to the percentage of species that show the expected direction of deregulation when using degenerate primers. Genes are listed according to percentage success and predicted direction of deregulation. * *p* < 0.05 when significance is tested against own species treated only with DMSO. Log2 fold change values are color-coded in both tables to easily show direction and magnitude of deregulation; red values indicate upregulation, and blue values indicate downregulation. Md: medaka; Zf: zebrafish; Tl: tilapia; Tl_1X: tilapia 1X concentration; Tl_1.5X: tilapia 1.5X concentration; Gp: guppy; ND: not detected.

To test the suitability and universality of these biomarker genes, we selected a third fish species of a different lineage: Mozambique tilapia (*Oreochromis mossambicus*), a member of the Cichlidae family. Mozambique tilapia were acutely exposed to PCB126. Since tilapia fingerlings were larger in size, toxicity treatment was carried out at 150 nM and 225 nM, i.e., 1X or 1.5X the concentration used for zebrafish and medaka. To validate potential PCB-126-induced biomarker genes, nine common DEGs genes identified from medaka and zebrafish, including seven upregulated genes (*cyp1b1*, *cyp1c2*, *cyp1a*, *cmbl*, *prdx1*, *hsp90aa1.2*, and *serpinh1b*) and two downregulated genes (*slc25a48* and *angptl2b*), were selected for validation in PCB126-treated tilapia by RT-qPCR using tilapia-specific primers (Supplementary Table S1) The results are shown in Figure 4A, alongside corresponding medaka and zebrafish log_2_ fold change values derived from RNA-seq analysis. *cyp1b1*, *cyp1c2*, and *cyp1a* are cytochrome P450 enzymes; a protein family known to metabolize various PCB toxins [51]. Upon PCB exposure in both mice and zebrafish, increased expression of *cyp1a* and *cyp1b1* has been previously observed in various tissue types, such as liver, lung/gills, and heart, while *cyp1c2* has shown increased expression in the zebrafish liver [52,53]. *prdx1*, *hsp90aa1,* and *serpinh1* are molecular chaperones that have also been reported in PCB-response studies. Primary murine astrocytes exposed to a PCB cocktail showed increased expression of *prdx1* [54]. In contrast to our data, *hsp90aa1* and *serpinh1* showed downregulation in zebrafish larvae and largescale sucker livers, respectively, when exposed to PCBs [55,56]. *cmbl*, *slc25a48*, and *angptl2*, an esterase, mitochondrial transporter, and angiopoietin-like protein, respectively, have not previously been implicated in PCB response or AhR pathway activation, although another SLC25 protein family member, *slc25a43*, showed increased expression in the adipose tissue of ringer seals exposed to a PCB-contaminated water source [57]. Considering that PCB toxins are known carcinogens, it is perhaps of interest that almost all tested genes, with the exception of *cyp1c2*, are reported to be either directly involved in or show deregulated gene expression in various cancers [58,59,60,61,62,63,64,65]. Of these nine genes tested, eight transcripts were detected, and all showed the same direction of deregulation as in medaka and zebrafish. Moreover, seven genes showed a greater extent of deregulation in the 1.5X concentration compared to the 1X concentration, suggesting that these genes are indeed deregulated in a concentration-dependent manner during PCB126 exposure; thus, they may be suitable biomarkers for PCB126 exposure.

To further assess the potential of the candidate genes as universal biomarkers more broadly for teleost fish, the guppy (*Poecilia reticulata*) was also chosen as the fourth fish species to validate the biomarker genes. The species diverged from the medaka and tilapia common ancestor 60–80 million years ago and is closely related to the stickleback fish and Amazon molly [66]. As most fish species, including the guppy, have limited or no gene sequence information, we used degenerate PCR primers designed based on multiple sequence alignment from divergent fish species. These degenerate PCR primers were used to test for the biomarker genes in response to acute PCB126 exposure in the four teleost species: medaka, zebrafish, tilapia, and guppy. The degenerate primer sequences are provided in Supplementary Table S2.

The results of the RT-qPCR using degenerate primers are shown in Figure 4B. Among the nine potential biomarker genes tested, there was a variable success, as indicated the successful rates that ranged from 25% to 100% for the four fish species, as shown in Figure 4B. *cyp1a* and *cyp1c2* were the most robust and showed consistently significant upregulation in all four species; thus, they are strong candidates as universal PCB126 biomarkers in teleost fish. *cyp1b1*, *serpinh1b*, *angptl2b,* and *slc25a48* showed anticipated deregulation in three out of four species, with *cyp1b1* and *angptl2b* showing more robust changes than the other two. *hsp90aa1.2* and *cmbl* both showed the expected upregulation in 50% of the species, while *prdx1* is the weakest candidate biomarker, showing upregulation in only one of the four species.

The two validation experiments clearly indicated that *cyp1a* and *cyp1c2* are the strongest and most consistent candidate biomarkers for PCB126 toxicity based on the tests in these four teleost fish. The cytochrome P450 family of monooxygenases represent the key proteins involved in the breakdown and metabolism of co-planar poly-aromatic hydrocarbons, specifically dioxins or dioxin-like compounds such as 2,3,7,8-tetrachlorodibenzodioxin (TCDD) and other PCBs, through the aryl hydrocarbon receptor (AhR) pathways [67,68]. As the activation of *cyp1a* and *cyp1c2* is well known to respond to these various AhR agonists, the current data clearly indicated that PCB126 falls into the category of AhR agonists. Clearly, additional biomarker genes targeting other biological pathways need to be identified for predicting specific contaminant chemicals. Though *cyp1b1*, along with *cyp1a* and *cyp1c2*, is part of this family and has been found to be increased in response to PCB exposure in mouse, rat, and human hepatocytes [44,69,70], degenerate primer validation did not show the expected upregulation in zebrafish. Multiple genes in all four species also did not show the anticipated deregulation despite significant RNA-seq analysis results and successful validation in tilapia using tilapia-specific primers. This suggests that it is likely that the degenerate primers for some of the genes did not have the same efficiency or predicted binding site across all four species. Degenerate primer design would have to be refined and improved in order to successfully target genes across multiple teleost fish. However, though PCB126 shows different sensitivity in medaka and zebrafish larvae [71], most genes tested in our study showed the predicted deregulation direction in at least three out of four species. Although there was variable success in detecting biomarker gene expression in these four fish species by using degenerate PCR primers, the success rates could be improved with refined and optimized degenerate primers and PCR conditions.

## 4. Conclusions

**Figure 5:**
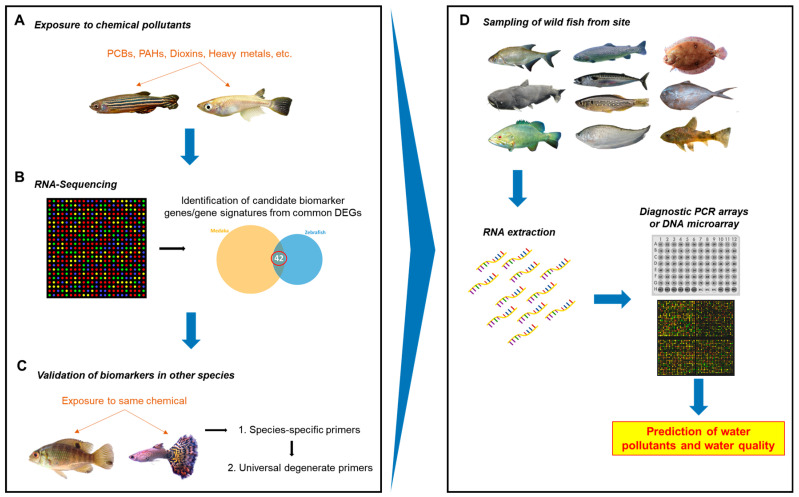
Roadmap to potential diagnostic method for onsite water quality monitoring. (**A**) Two laboratory fish species of distant evolutionary lineages are exposed to a toxic environmental pollutant chemical. (**B**) The fish are harvested for RNA-seq analyses. Candidate biomarker genes or gene signatures are identified from the list of commonly deregulated genes. (**C**) A two-step validation is then performed for the candidate genes: first using species-specific primers and then using universal degenerate primers. Strong candidate biomarkers and/or a biomarker signature are identified from the validation steps. (**D**) Downstream, diagnostic tools can be developed using strong biomarkers and/or signatures identified for various classes of chemicals. Wild fish could be collected from the water site and RNA extracted. Rapid PCR array and/or DNA array could be developed for multiple biomarker genes and used to predict the presence of particular classes of chemical pollutants.

In this study, we showed that candidate universal biomarker genes upon chemical exposure could be identified and validated in multiple evolutionarily distant teleost species via transcriptome analysis and RT-qPCR. Our study indicated a possible roadmap, as illustrated in Figure 5, to use the method presented in this paper to identify common biomarker genes for each toxicant chemical and to develop molecular tools for analyzing biomarker gene expression from on-site fish specimens collected from diverse wild waters in order to discover potential toxicant contamination and their potential biological effects.

First, the two well-established laboratory fish models with well-annotated genome sequences, the zebrafish and medaka, can be used to conduct exposure experiments with a toxic chemical or class of chemicals (Figure 5A). Since the liver is the main organ for drug metabolism, liver tissue can be harvested and used for RNA sequencing. Potential biomarkers can be identified from the list of deregulated genes that are commonly found between the two species, based on genes that are known to be involved in chemical response and high and/or easily-detectable levels of expression (Figure 5B). Validation via RT-qPCR can be conducted with additional fish species using species-specific primers to ascertain whether the genes are suitable as candidate biomarkers and. if possible, to identify a biomarker signature. Finally, degenerate primers that are non-species-specific can be designed and tested in more fish species with known or unknown gene sequence information (Figure 5C). However, primers will need to be designed to bind more specifically and with high efficiency as well as validated thoroughly in order to maximize true positives and minimize false negatives. Eventually, biomarker data from tests of various pollutants or classes of pollutants can be used to design a diagnostic PCR array or DNA microarray that will allow for testing any sampled wild fish species from any water system (ocean, lakes, rivers, reservoirs, etc.) to predict water contamination (Figure 5D).

## Data Availability

The transcriptomics data generated and analyzed during the current study are available from the corresponding author, upon reasonable request.

## Supplementary Materials

The following supporting information can be downloaded at: https://www.mdpi.com/article/10.3390/cimb46080466/s1, Supplementary Table S1. Tilapia-specific primer sequences; Supplementary Table S2. Degenerate primer sequences.
