# Supplementary material for: Identifying Universal Fish Biomarker Genes in Response to PCB126 Exposure by Comparative Transcriptomic Analyses
Source: Curr Issues Mol Biol. 2024 Jul 23;46(8):7862–76. doi: 10.3390/cimb46080466 (PMC11352907; doi:10.3390/cimb46080466)
Supplement: Supplementary file 1 [file cimb-46-00466-s001.zip › cimb-3044150-supplementary.pdf]

Supplementary Table 1. Tilapia-specific primer sequences

Tilapia Ensembl ID	Gene Name	Tilapia-specific primer sequence		Product length (bp)
		Forward (5'→3')	Reverse (5'→3')	
ENSONIG00000010908	*angptl2b*	AGCAGATCCAACAAATTGCGT	GCCTACTCGCTGACGTTACT	152
ENSONIG00000014355	*cmbl*	GACATGCTTGCTGCTAACGG	GGCTTCAAGAGCACTCACCT	387
ENSONIG00000002935	*cyp1a*	ATGAGCAAACGCTACGGTGA	CTGGGGTTGTGCCCTCTAAG	265
ENSONIG00000010388	*cyp1b1*	ACACATTTACAGCAGGCGGA	GCTGATTACAACAGAGCCAACT	404
ENSONIG00000021032	*cyp1c2*	CCCCTGATGTGGAAAGACCC	ACTGGTGCAGAAGAATCGCA	181
ENSONIG00000000805	*hsp90aa1.2*	TTCCACTTGCGTTGACCACA	GGTAACCCACCTGTCCAATCT	302
ENSONIG00000018611	*prdx1*	TCCCACTTCTCCCATTTCGC	GGTCTCCTCAACAGAGCGTC	231
ENSONIG00000005220	*serpinh1b*	AGCCATCTCCCTGCCTAAGA	ACCGAAGATGCTTGTGTCGT	211
ENSONIG00000004280	*slc25a48*	TTTCAACACAAGCAGGGGGT	CGAGCCTATACAGCTTGGAGT	170
ENSONIG00000008505	*actb*	GACCCACACAGTGCCCATCT	TCTCGGCTGTGGTGGTGAA	140

Supplementary Table 2. Degenerate primer sequences

Gene Name		Degenerate primer sequence (5'→3')	Primer length (NT)	Degeneracy	Clamp score	Primer amino acid sequence	Product length (bp)
angptl2b	F:	GCGCTCACTACCAGAAGGGAggntggtggta	31	4	82	CAHYQKGGWWY	186
	R:	AATGTGTTAGGGTTTGGTCGdatcatcatnac	32	12	78	VMMIRPNPNTF	
cmbl	F:	GGCAGCTGCCTAACACACGAtayatggcnga	31	8	76	WqLPNTRYMAD	258
	R:	TAGTGGGTAGCCACTCCTCCccarcaraancc	32	16	77	GFCWGGVATHY	
cyp1a	F:	AACGTGATCTGCGGAatgtgyttygg	31	4	79	VANVICGMCFG	302
	R:	CTCGTCCAGCTTTCGrtcytcrcartg	32	16	78	HCEDRKLDENS	
cyp1b1	F:	GGATCCATCGTGGACgtnatgccntg	26	16	74	GSIVDVMPW	182
	R:	AGCCACGATGAAAGCrtcngtcatrtc	27	16	73	DMTDAFIVAL	
cyp1c2	F:	GCTATGCAGCTGGGAcaratgccnca	26	8	83	AMQLGQMPH	566
	R:	AGGGAAGGACTGCAGccanggcatnac	27	16	74	VMPWLQSFPN	
hsp90aa1.2	F:	AGCACAACGACGACGAGCAGtaygcntggga	31	8	83	KHNDDEQYAWE	144
	R:	CGCTCCTCGAGGTACTCGGTytgrtcytcytt	32	16	79	KEDQTEYlEER	
prdx1	F:	ACAGAGGAAAGTACGTGGTGttyttyttyta	31	8	78	YRGKYVVFFFY	288
	R:	AGTCCTCGGTAAGCGATTCCytcrtcytcytt	32	16	76	KEDEGIAYRGL	
serpinh1b	F:	TCGTGAAGAACTCCAAGAAGcaytayaayta	31	8	74	FVKnSKKHYNY	204
	R:	AACTTCTCGTCCCAGTGAGGyttraaraacat	32	8	81	MFFKPHWDEkF	
slc25a48	F:	ACCGTGACCGGATTCttyaarggnat	26	16	72	TVaGFFKGM	254
	R:	AGCCAGCACCGTCTGngtytgcatytg	27	16	74	QMQTQmVLAE	
actb	F:	GGAGTGATGGTGGGAATGGGNCARAA	26	8	–	GVMVGMGQ	137
	R:	GTGCCAGATCTTCTCCATRTCRTCCCA	27	4	–	VPDLLHXXP	
